# Pseudorabies virus infection (Aujeszky’s disease) in an Iberian lynx (*Lynx pardinus*) in Spain: a case report

**DOI:** 10.1186/s12917-016-0938-7

**Published:** 2017-01-05

**Authors:** A. Javier Masot, María Gil, David Risco, Olga M. Jiménez, José I. Núñez, Eloy Redondo

**Affiliations:** 1 Unidad de Histología y Anatomía Patológica, Departamento de Medicina Animal, Facultad de Veterinaria, Universidad de Extremadura, Avda. Universidad s.n., Cáceres, 10003 Spain; 2 Veterinary Teaching Hospital, University of Extremadura, Cáceres, Spain; 3 Innovación en Gestión y Conservación de Ungulados S.L., Cáceres, Spain; 4 Dirección General de Medio Ambiente, Consejería de Medio Ambiente y Rural, Junta de Extremadura, Spain; 5 Institut de Recerca i Tecnologia Agroalimentàries (IRTA)-Centre de Recerca en Sanitat Animal (CReSA), UAB-IRTA, Campus de la Universitat Autònoma de Barcelona, Bellaterra, Cerdanyola del Vallès Spain

**Keywords:** Iberian lynx, *Lynx pardinus*, Pseudorabies virus, Aujeszky’s disease, Suid Herpesvirus 1, Endangered species, Case report

## Abstract

**Background:**

The only natural hosts of Pseudorabies virus (PRV) are members of the family Suidae (*Sus scrofa scrofa*). In species other than suids infection is normally fatal. In these mammals, including carnivores, PRV typically causes serious neurologic disease. The endangered Iberian lynx (*Lynx pardinus*) is a wild feline endemic to south-western Europe (Iberian Peninsula). The Iberian lynx was found to be the world’s most endangered felid species in 2002. In wild felines, PRV infection has only been previously reported once in a Florida panther in 1994. No seropositive lynxes have ever been found, nor has PRV been detected in dead Iberian lynxes to date.

**Case presentation:**

We describe the first reported case of pseudorabies in an Iberian lynx (*Lynx pardinus*). Pseudorabies was diagnosed in a young wild Iberian lynx from Extremadura (SW Spain) by histopathological examination, immunohistochemistry, polymerase chain reaction (PCR) and sequence analysis. Gross lesions included alopecia of the ventral neck, bloody gastro-intestinal contents and congestion of the brain. Histopathological analysis showed a moderate nonsuppurative meningoencephalitis with diffuse areas of demyelination, necrotizing gastritis and enteritis of the small intestine. Pseudorabies virus (PRV) antigen was found in neuronal and non-neuronal cells of the brain, tonsils, and gastric glandular epithelial cells by immunohistochemical analysis. The presence of the virus in the brain was confirmed by nested PCR. The sequence analysis of the 146 bp fragment (from the viral glycoprotein B gene) showed that the amplified sequence matched (with 100% identity) the PRV genome. Furthermore, specific DNA from glycoprotein D and E encoding-genes was detected by conventional and real-time PCR, respectively, confirming the latter that this infection was produced by a wild-type PRV strain.

**Conclusions:**

This study supports the suspicion that PRV could infect the Iberian lynx. The detection of PRV in a dead Iberian lynx suggests that the virus may have a negative impact on the survival of endangered lynxes in the wild. However, because this is the first verified instance of lynx mortality resulting from pseudorabies, its true impact on the population is unknown.

**Electronic supplementary material:**

The online version of this article (doi:10.1186/s12917-016-0938-7) contains supplementary material, which is available to authorized users.

## Background

The Iberian lynx (*Lynx pardinus*) is an endangered wild feline endemic to the Iberian Peninsula, located in the southwest corner of Europe. The Iberian lynx population suffered a dramatic reduction in size during the 20^th^ century [1]. From 2002 to 2015 it was considered the world’s most endangered wild feline species. Recently, the Iberian lynx has been reclassified from a critically endangered to an endangered species [2]. The population was 406 at the 2015 census [3]. Iberian lynxes predominantly prey on wild rabbits (*Oryctolagus cuniculus*), although they alternatively may prey on small mammals, juvenile ungulates, partridges, other birds, reptiles and insects [4]. Moreover, Iberian lynxes occasionally consume wild boar as prey and as carrion, which puts them at risk for pseudorabies infection [5]. The species is highly solitary and territorial, although family groups have occasionally been detected [6]. The Iberian lynx is found in areas of Mediterranean scrub and in habitats of open forests and thickets with high rabbit abundance [7]. The small population and low genetic diversity of the Iberian lynx make this species especially vulnerable to bacterial and viral infections. A number of infectious agents have been detected in Iberian lynxes, including a variety of feline viral and bacterial pathogens [8, 9].

The causative agent of Aujeszky’s disease (AD) is Suid Herpesvirus 1 (SuHV1), which is also known as pseudorabies virus (PRV). This virus is a member of the genus *Varicellovirus* belonging to the subfamily *Alphaherpesvirinae* of the family *Herpesviridae* [10] that can infect a wide range of species, though it is does not affect the higher primates [5]. Pseudorabies is a viral disease which was first reported in dogs in 1902 [11], and is prevalent throughout the world. The only natural hosts of PRV are domestic and wild suids (*Sus scrofa scrofa*) and their hybrids [12]. Symptoms of AD depend on the age of the animal, and can include respiratory, reproductive and nervous signs [13]. PRV is a neurotropic virus which replicates in the nasopharyngeal mucosa, before taking a number of nervous pathways to reach the central nervous system (CNS). It results in a non-suppurative meningoencephalitis which is frequently fatal in piglets [14–17]. PRV seroprevalence in European wild boar varies considerably between different geographic regions. The highest seroprevalences were described in Mediterranean countries including Spain (up to 100%) [5, 12, 18–24]. In both domestic pigs and wild boar, the disease is usually subclinical because of the virus-host adaptation; however, in piglets, the infection is commonly fatal [15–17]. Like other herpesviruses, PRV usually produces latent infections in the host. PRV can infect the neurons and glial cells of apparently healthy swine over long periods. These latent infections can be reactivated, which means PRV can spread to other susceptible animals, mainly mammals, such as carnivores [14, 25], although humans seem insusceptible [26]. Species other than suids can be considered dead-end hosts because the disease is generally fatal before the virus is excreted [5]. In these mammals, including carnivores, PRV infections usually result in very severe neurological symptoms which often involve localised pruritus [26], resulting in death within hours after appearance of the first symptoms [27]. There are considerable implications for conservation, as cases have been reported in endangered carnivores such as the Florida panther [28], the wolf [25] and captive brown bears [29] after consumption of PRV-contaminated meat. No seropositive lynxes have ever been found, nor has PRV been detected in dead Iberian lynxes to date.

This article is the first reported case of pseudorabies in an Iberian lynx. We describe the second reported case of pseudorabies in a wild feline considering that PRV infection has only been previously reported once in a Florida panther in 1994 [28].

PRV infection was suspected in this lynx based on histopathological findings, and because PRV infections in wild boar are endemic in SW Spain. Infection was confirmed with immunohistochemistry, PCR, and sequence analysis.

## Case presentation

The lynx that we studied was a wild ~9-month-old male, born to a healthy 3-year-old dam. It belonged to the first two litters born in Extremadura (SW Spain) after *Lynx pardinus* was re-established in this region through the LIFE+ 10NAT/ES/ 000570 project. Using camera trapping, the estimated date of its birth was established to have been between March 8 and 12, 2015.

This lynx was captured, subjected to a routine sanitary evaluation protocol, radio-collared, vaccinated against Feline Leukemia Virus (PureVax FeLV, Merial, Barcelona; Spain) and relocated back into the wild on Nov. 18, 2015. A complete blood count and plasma protein tests showed normal levels. Plasma biochemistry results were within normal ranges, although plasma concentrations of glutamyl transpeptidase and creatine phosphokinase were slightly increased. PCR tests were negative for infection with Feline Leukemia Provirus (FeLV), Feline Immunodeficiency Virus (FIV) and Canine Distemper Virus (CDV) in the blood; Feline Calicivirus (FCV) and Feline Herpesvirus (FHV1) in oropharyngeal swabs; and Feline Coronavirus (FCoV) and Feline Parvovirus (FPV) in rectal swabs. The lynx was found to be antigen-ELISA (enzyme-linked immunosorbent assay) negative for FHV1, FCoV and CDV as well as negative for FCV and FPV by fluorescent antibody testing. These tests were performed as previously described [8, 30, 31]. Finally, a blocking ELISA test (CIVTEST SUIS ADV gE, Hipra, Gerona, Spain) was used to detect the presence of serum antibodies against PRV, obtaining negative results.

The lynx was found dead on Dec 1, 2015 on private land consisting of a mixture of dense scrub and open pasture in an area known as “Hornachos-Valle del Matachel” located southwest of Badajoz (Extremadura), Spain (Latitude: 38°27′10.98″ N, Longitude: 5°54′30″W).

**Fig. 1:**
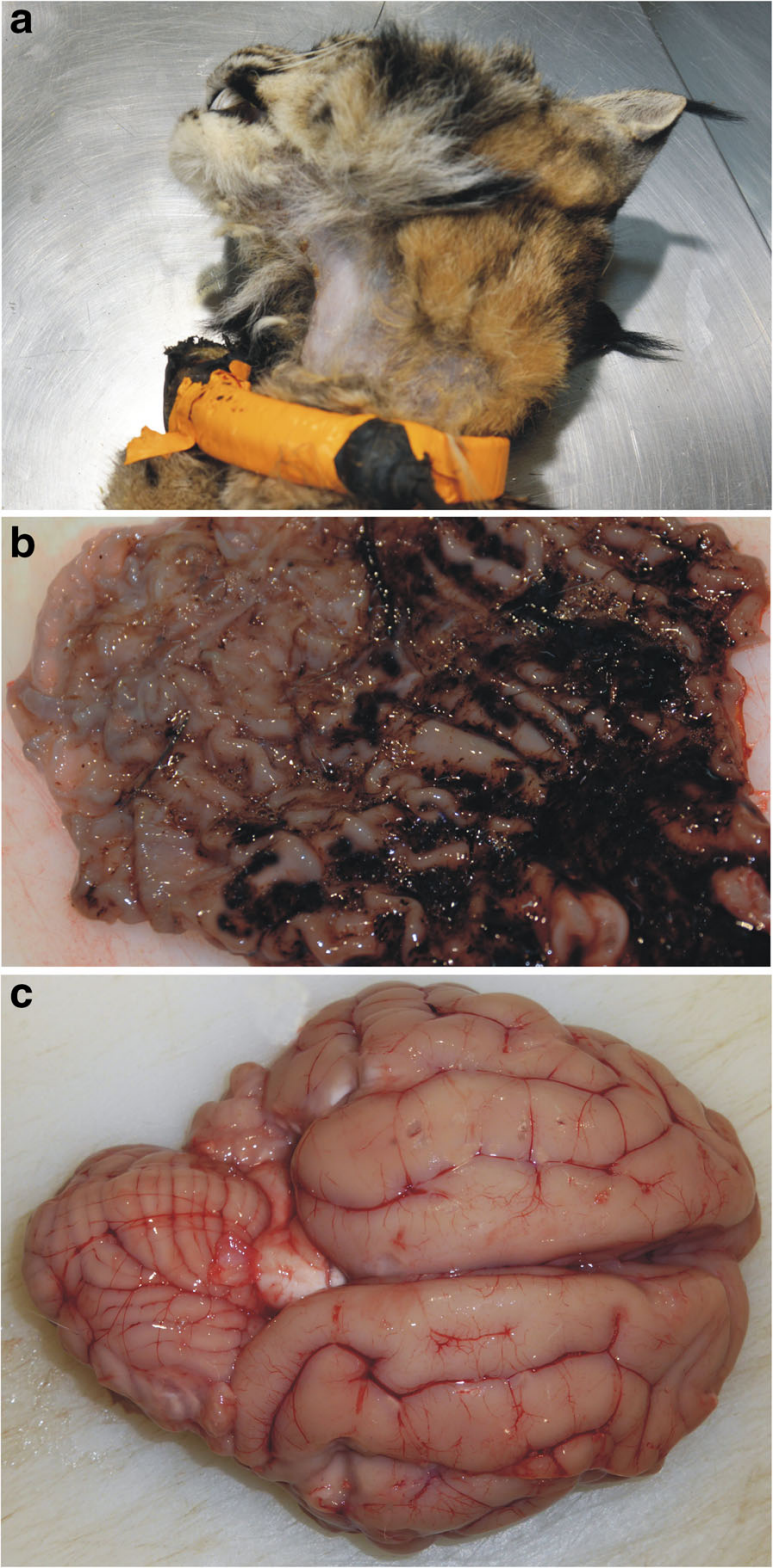
Gross pathology findings. Iberian lynx. **a** Area of alopecia on the ventral neck skin. The radio-collar appears scratched. **b** Partially digested blood was present in the lumen of the stomach. **c** Congestion of the brain

Post-mortem examination was carried out at the Veterinary Teaching Hospital of Extremadura (Cáceres, Spain). Upon presentation for necropsy, the lynx weighed 3,060 g, and the carcass was preserved without putrefaction changes. An X-ray examination excluded general traumatisms or the presence of shotgun wounds. Gross lesions of the lynx were minimal. In agreement with our observations, AD in many cases does not develop significant macroscopic lesions in other carnivores such as dogs and cats [32, 33] and wolves [25]. The skin of the ventral neck was denuded of hair and the radio-collar appeared torn (Fig. 1a). Intense pruritus can sometimes lead to these types of lesions due to scratching and self-mutilation as has been suggested in coyotes [34], dogs [26, 35] and cats [32]. The stomach and small intestine contained a moderate amount of partially digested blood (Fig. 1b). The large intestinal contents consisted of varying amounts of dark red to black semi-formed fecal material. The meninges were congested (Fig. 1c). These lesions are similar to those reported in the Florida panther [28], coyotes [34] and dogs [11].

**Fig. 2:**
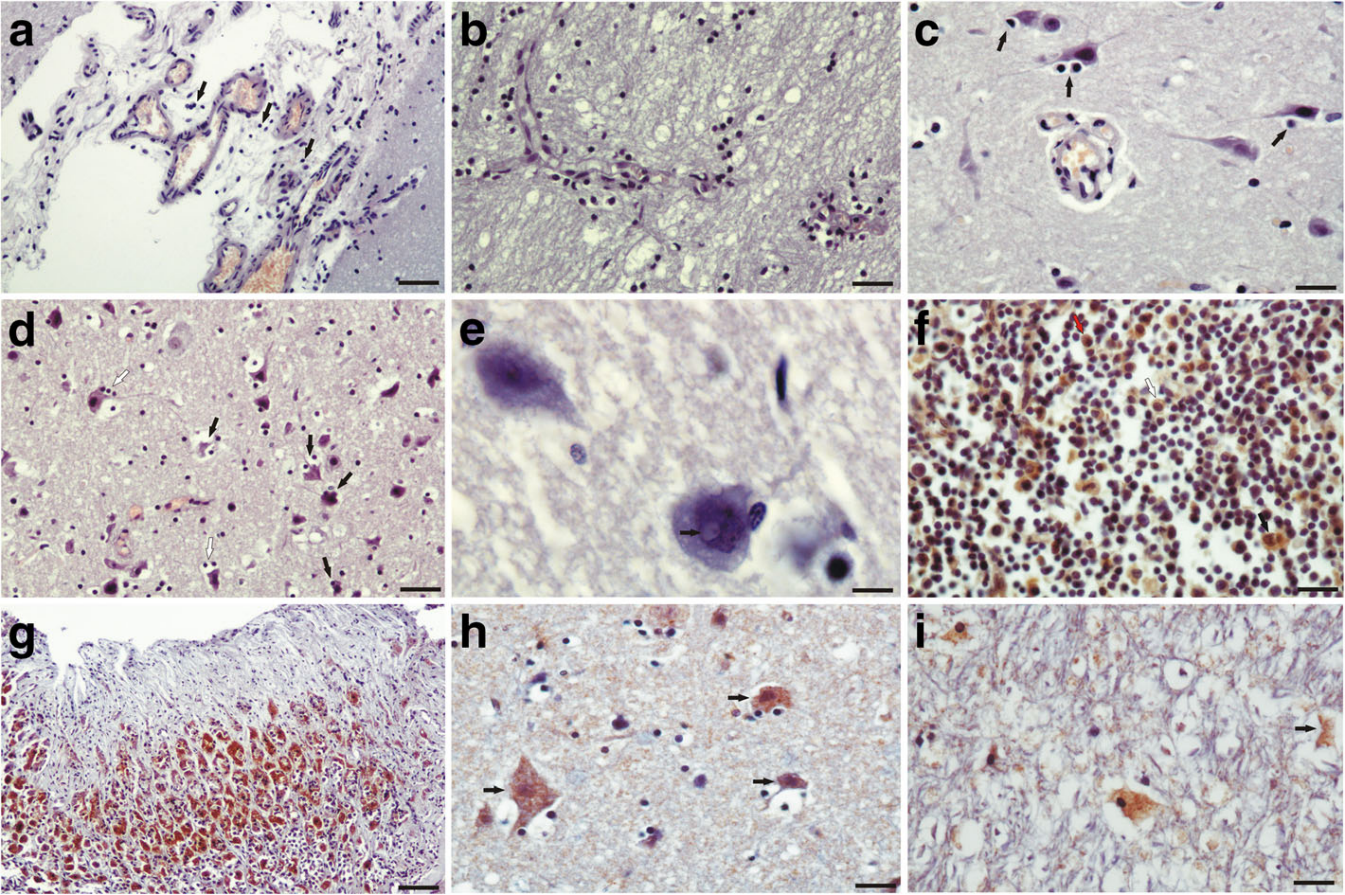
Histopathology and immunohistochemistry. Iberian lynx **a** Cerebrum. The leptomeninges and subarachnoid space were infiltrated by mononuclear cells (*arrows*) HE (bar = 25 μm). **b** Cerebrum. Mononuclear cellular infiltrates around blood vessels and neuropil. Demyelination and spongiosis. HE. (bar = 35 μm). **c** Cerebrum. Glial satellitosis (*arrows*). HE. (bar = 40 μm). **d** Cerebrum. Neuronal necrosis and neuronophagia (black arrows). Satellitosis (*white arrows*). HE. (bar = 35 μm). **e** Cerebrum. Neuronal intranuclear inclusion bodies (*arrow*). HE. (bar = 50 μm). **f** Tonsils. Positive immunostaining for PRV antigen in lymphocytes (*white arrow*), plasma cells (*red arrow*) and macrophages (*black arrow*). PDM. DAB chromogen. (bar = 40 μm). **g** Stomach. PRV antigen was detected in gastric glandular epithelial cells. PDM. DAB chromogen. (bar = 35 μm). **h** Cerebrum. Immunohistochemical PRV detection in neurons (*arrows*). PDM. DAB chromogen. (bar = 40 μm). **i** Cerebellum. Immunohistochemical demonstration of PRV presence in glial cells of white matter of the cerebellum. Some cells are necrotic (*arrow*). PDM. DAB chromogen. (bar = 40 μm)

Representative portions of sampled tissues were fixed in 10% neutral buffered formalin, routinely embedded in paraffin and hematoxylin and eosin (HE) stained. A histopathological analysis of the CNS showed diffuse nonsuppurative meningoencephalitis similar to that reported in domestic cats [32, 36] and other unnatural hosts such as dogs [11, 33, 35, 37], foxes [38] and coyotes [34]. Similar to that described for coyotes [34], the leptomeninges and subarachnoid space were infiltrated and expanded by slight perivascular accumulations of mononuclear cells (Fig. 2a). This meningoencephalitis was characterized by mononuclear cellular infiltrates around blood vessels (perivascular cuffs) and neuropil (Fig. 2b) composed mainly of lymphocytes, as well as multifocal to diffuse microgliosis, perineuronal glial satellitosis (Fig. 2c and d), neuronal necrosis and neuronophagia (Fig. 2d). Most neurons appeared unaffected; although within damaged brain regions, several neurons showed eosinophilic intranuclear inclusion bodies (Fig. 2e), even though eosinophilic intranuclear inclusions could be absent in the neurons of cats [39]. Diffuse areas of demyelination and malacia were observed in sections of the cerebrum and cerebellum (Fig. 2b). These lesions have been previously described in raccoons [40]. Gastrointestinal tract lesions observed in the lynx consisted of necrotizing gastritis and enteritis of the small intestine with foci of epithelial necrosis with minimal inflammatory reactions. These lesions have been reported in cats and dogs [36, 41] and in piglets [42].

PCR tests were negative for infection with FCoV (clot, spleen, mesenteric ganglia, small intestine and intestinal scraping samples), FCV (clot samples), CDV (brain tissue, clot, mesenteric ganglia and intestinal scraping samples), FPV (clot, mesenteric ganglia and intestinal scraping samples), FHV1 (clot, spleen, mesenteric ganglia, small intestine, intestinal scraping and brain tissue samples), FIV (clot and spleen samples), FeLV provirus (clot, mesenteric ganglia, intestinal scraping and brain tissue samples), *Mycoplasma haemofelis*, *Candidatus mycoplasma haemominutum*, *Candidatus mycoplasma turicensis*, *Anaplasma phagocytophilum*, *Bartonella henselae*, *Chlamydophila felis* [8], *Cytauxzoon felis* (clot samples) [43] and *Leptospira spp.* (kidney samples) [44]. PCR tests were positive for infection with FeLV provirus (spleen and bone marrow samples) [8] and *Pasteurella spp.* (lung samples) using PrimerDesign™ genesig Kit for Pasteurella multocida (Genesig, Chandler’s Ford, United Kingdom). Toxicological analyses were negative for pesticides and other organic compounds (chloralose, barbiturates, and metaldehyde), anticoagulant rodenticides and anticholinesterase pesticides. No intestinal parasites were detected in the lynx feces.

**Fig. 3:**
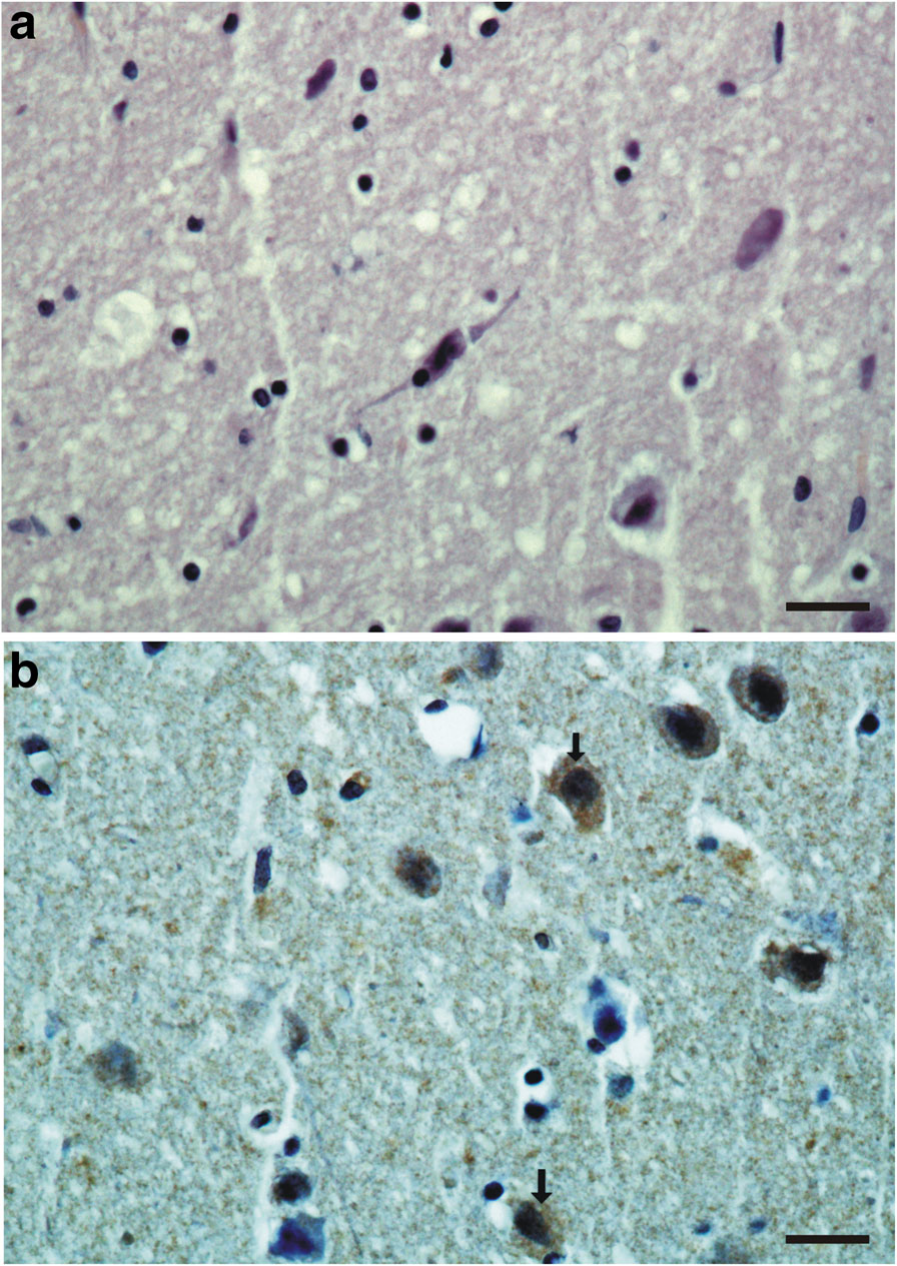
Immunohistochemistry. **a** Iberian lynx. Cerebrum. Negative control for immunohistochemical PRV detection by substitution of the primary antibody with normal mouse serum (1:100). PDM. DAB chromogen. (bar = 40 μm). **b** PRV infected swine. Brain. Positive control for immunohistochemical PRV detection. PDM. DAB chromogen. (bar = 35 μm)

The polymer detection method (PDM) to detect porcine PRV was carried out on deparaffinized tissue sections using an UltraVision Quanto Detection System HRP DAB (Thermo Scientific, Fremont, USA, #TL-060-QHD) following manufacturer’s directions. Primary antiserum was gE PRV-specific monoclonal antibody (Ingenasa, Madrid; Spain #M.11.ADV.B2CF2). In order to determine the specificity of the immunohistochemical reaction, primary antibody was replaced with PBS or with non-immune mouse serum (1:100). The positive control consisted of a slide containing known positive tissue (CNS of naturally PRV infected pig). Negative control slides consisted of brain sections of a PRV-free lynx. The PRV antigen was detected in the tonsils (Fig. 2f) of the Lynx. PRV has been previously detected by immunohistochemistry in the tonsils of pigs [45] and it was isolated from de tonsils of dogs [33] and racoons [40]. The replication of the virus in tonsils after its entrance via the oral route has been described in cats [32]. Positive immunostaining was also observed in numerous gastric glandular epithelial cells (Fig. 2g), consistent with that previously described in dogs [37]. The PRV antigen was found in the cytoplasm of neuronal (Fig. 2h) and non-neuronal cells (Fig. 2i) in inflamed brain areas. This localization has been described in dogs [11, 37]. Like other viruses belonging to the subfamily *Alphaherpesvirinae*, PRV first replicates in the epithelium of the mucous membranes, and then reaches the CNS via the nerve fibres that innervate the colonised tissues [46]. Specific staining was not detected in the negative control samples (Fig. 3a). The positive control, showed positive immunoreaction against the gE PRV-specific monoclonal antibody (Fig. 3b).

**Fig. 4:**
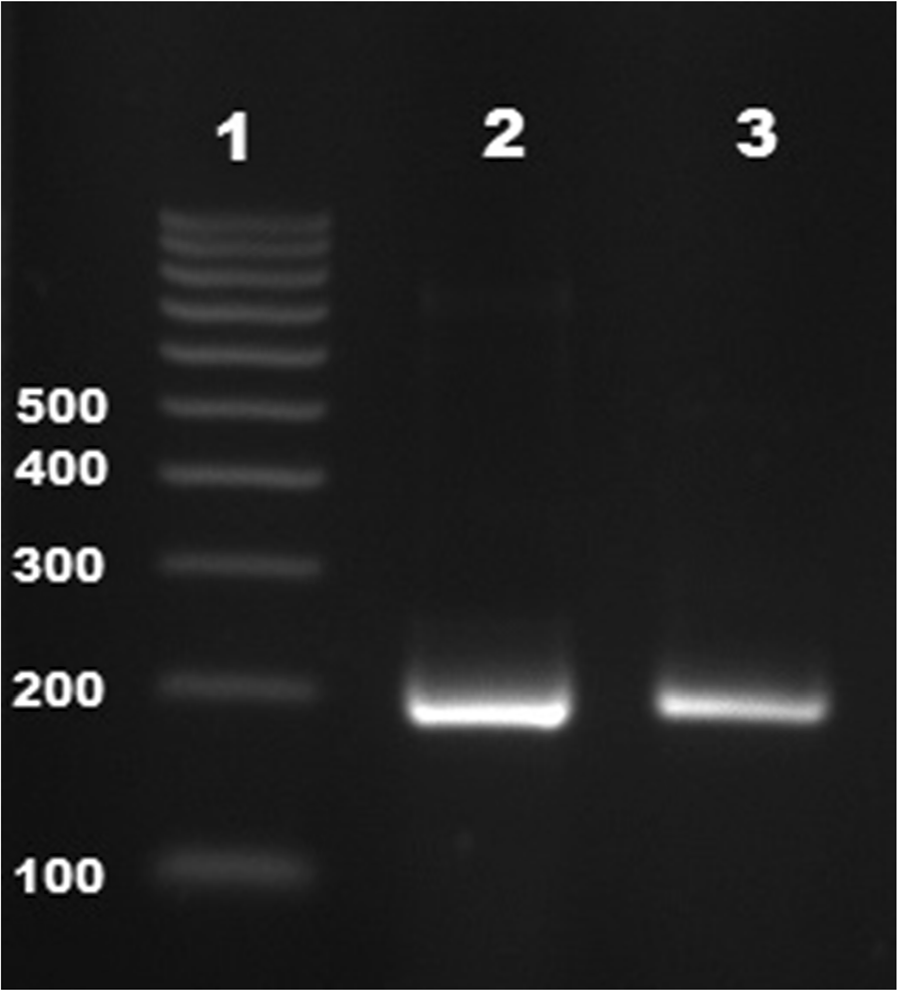
Agarose gel electrophoresis of Suid Herpesvirus 1 (SuHV1) from nested PCR (gB gene) stained with ethidium bromide. Line 1: 100 bp DNA ladder. Line 2: 195 bp band obtained from a positive control of SuHV1 (strain Bartha K-61). Line 3: 195 bp band obtained from DNA extracted from brain tissue of the studied Iberian Lynx

To assess the presence of PRV in the CNS of the affected animal, a specific nested PCR reaction (Fig. 4) designed to amplify the viral glycoprotein B gene was performed, using two pairs of primers previously described [47]. DNA from brain tissue was obtained using a commercial kit (QiAmp DNA Mini Kit®, Qiagen Ltd., Crawley, West Sussex, UK) following the manufacturer’s protocol. A total of 5 μl of the extracted DNA was used as a template for the nested PCR in a 25 μl reaction mixture containing 12.5 μl of PCR Master Mix (2x) (Green Taq, Thermo Fisher Scientific, Waltham, MA, USA) and 0.2 μM of each primer. DNA was amplified using the following amplification procedure: 1 cycle at 95 °C for 3 min, 30 cycles of denaturalization (94 °C, 45 s), annealing (62 °C, 1 min) and extension (72 °C, 1 min); and a final extension at 72 °C for 10 min. The second step of the nested PCR was performed using 0.5 μl of the first PCR product as template and following the amplification procedure previously described. PCR products were separated by 2% agarose gel electrophoresis and visualized by ethidium bromide staining under UV light.

The obtained 195 bp band was cut out of the gel, and DNA was extracted using an Ultra Clean™ GelSpin DNA Purification Kit (Mo Bio Laboratories, Inc., Carlsbad, California, 92010, USA). The 195 bp amplified product was sequenced in both directions using a BigDye Terminator v3.1 cycle sequencing kit according to the manufacturer’s instructions (Applied Biosystems). Nucleotide sequences were read on a 3730xl DNA Analyzer (Applied Biosystems). After trimming sequence primers, the 146 bp sequence was initially compared with GeneBank sequences using the Basic Local Alignment Search Tool (BLAST) (http://www.ncbi.nlm.nih.gov/blast/Blast.cgi) to verify the identity of the fragment. The BLAST analysis showed that the 146 bp sequence matched with the PRV genome. The obtained sequence and PRV available sequences were aligned by Clustal Omega [48]. The alignment showed that the analyzed fragment presented 100% sequence identity with reference PRV strains such as Kaplan or Bartha. One substitution was found in strains such as NIA3 or Becker, while 2–4 substitutions were found when isolates with an Asian origin were aligned, like HUYD (DDBJ accession number KJ526432) or Rang (DDBJ accession number KP895102) isolates. An additional PCR was carried out to amplify glycoprotein D gene with primers and conditions previously described [49]. Furthermore, in order to assess whether PRV infection was produced by a wild-type or a vaccine PRV strain, a TaqMan® base real-time PCR assay for the detection of gE gene was conducted as previously described using DNA extracted from CNS as template [50]. Specific DNA from glycoprotein D and E encoding-genes was detected confirming the latter that this infection was produced by a wild-type PRV strain.

This article describes the first reported case of pseudorabies in Iberian lynxes, and it confirms that they are susceptible to PRV infection. Like other felids, PRV-infected lynxes develop lethal neurological disorders. In cats, PRV infection with brief two- to four-day incubation period, produces acute encephalitis and can cause 100% mortality in experimentally infected domestic cats [32, 51]. The cats suffer from anorexia, occasional severe itching with lesions caused by scratching and self mutilation and uncoordinated movements and paralysis [32, 51]. The outcome is invariably fatal and leads to death within 12–48 h of the first appearance of clinical signs [32]. This peracute death does not allow time for a serologic response to occur [52]. If Iberian lynxes succumb as rapidly as domestic cats, detection of the clinical phase can prove to be very difficult.

The main sources of infection for cats are uncooked pig or offal [32]. Direct spread of virus from infected to noninfected carnivores likely does not occur [34]. Wild boar are a well known reservoir for PRV and hence, may pose a risk to transmit the infection to wildlife carnivores species [25, 34]. Some authors have described recently, rates of 69.70% ELISA seropositivity and 11.30% of PRV lung infections in wild boar population throughout SW Spain [53, 54]. Wild boar are not the main component of the Iberian lynx diet; however, these animals do occasionally consume wild boar as prey and as carrion [5]. Therefore, it is conceivable that some lynxes will be exposed to PRV from the ingestion of wild boar. Indirect transmission can also occur through viral excretion by pigs, without direct contact with the pigs themselves [32]. The presence of wild boar infected with the PRV could have a negative impact on conservation of wild carnivores which consume wild boar [25] such as lynxes.

## Conclusions

Our findings support the suspicion that PRV could infect the Iberian lynx [5, 22]. The detection of PRV in a dead Iberian lynx suggests that the virus may have a negative impact on the endangered lynx’s survival in the wild. However, because this is the first verified instance of lynx mortality resulting from pseudorabies, its true impact on the population is unknown. A possibility for management of this species is vaccinating lynxes with an inactivated vaccine during routine captures to provide some level of immunity against this disease. This has previously been suggested for Florida panthers [28], although vaccination has not been proven efficacious. Oral vaccination of wild boar against classical swine fever virus has been shown to be successful [55], which means that oral vaccination of wild boar against PRV could be an effective strategy for protecting lynxes from the disease [25]. The effectiveness of using attenuated live vaccine for oral immunisation of wild boar against PRV has been demonstrated [56], though the safety of this technique needs to be studied in depth [25].

## Additional files

Additional file 1: Table S1. Primers used for SuHV1 amplification. (DOCX 90 kb)
Additional file 2: File S2. Partial glycoprotein B nucleotide sequence (SuHV1) isolated from *Lynx pardinus*. (DOCX 42 kb)

## Abbreviations

AD: Aujeszky’s disease
CDV: Canine Distemper Virus
CNS: Central nervous system
DAB: Diaminobenzidine
ELISA: Enzyme-linked immunosorbent assay
FCoV: Feline Coronavirus
FCV: Feline Calicivirus
FeLV: Feline Leukemia Virus
FHV1: Feline Herpesvirus
FIV: Feline Immunodeficiency Virus
FPV: Feline Parvovirus
HE: Hematoxylin and eosin
PCR: Polymerase chain reaction
PDM: Polymer detection method
PRV: Pseudorabies virus
SuHV1: Suid Herpesvirus 1
