# Supplementary material for: Pseudorabies virus infection (Aujeszky’s disease) in an Iberian lynx (Lynx pardinus) in Spain: a case report
Source: BMC Vet Res. 2017 Jan 5;13:6. doi: 10.1186/s12917-016-0938-7 (PMC5217549; doi:10.1186/s12917-016-0938-7)
Supplement: Additional file 1: Table S1. — Primers used for SuHV1 amplification. (DOCX 90 kb) [file 12917_2016_938_MOESM1_ESM.docx]

| **Primer name** | **Sequence (5‘-3‘)** | **Gene** | **Purpose** | **Position*** | **Reference** |
| --- | --- | --- | --- | --- | --- |
| gB first 05 | ATGGCCATCTCGCGGTGC | Gene B | Nested-PCR run 1 | 17870-17853 | [47] |
| gB first 03 | ACTCGCGGTCCTCCAGCA | Gene B | Nested-PCR run 1 | 17537-17554 | [47] |
| gB second 05 | ACGGCACGGGCGTGATC | Gene B | Nested-PCR run 2 | 17755-17739 | [47] |
| gB second 03 | GGTTCAGGGTCACCCGC | Gene B | Nested-PCR run 2 | 17561-17577 | [47] |
| ADV-GDA | cacggaggacgagctggggct | Gene D | PCR | 121576-121596 | [49] |
| ADV-GDB | gtccacgccccgcttgaagct | Gene D | PCR | 121792-121772 | [49] |
| gE694F | CTTCCACTCGCAGCTCTTCTC | Gene E | RT-PCR | 124258-124278 | [50] |
| gE765R | GTRAAGTTCTCGCGCGAGT | Gene E | RT-PCR | 124347-124329 | [50] |

*Position corresponds to Suid herpesvirus 1 strain Kaplan (DDBJ accession number JQ809328)
