# Supplementary material for: Pseudorabies virus infection (Aujeszky’s disease) in an Iberian lynx (Lynx pardinus) in Spain: a case report
Source: BMC Vet Res. 2017 Jan 5;13:6. doi: 10.1186/s12917-016-0938-7 (PMC5217549; doi:10.1186/s12917-016-0938-7)
Supplement: Additional file 2: File S2. — Partial glycoprotein B nucleotide sequence (SuHV1) isolated from Lynx pardinus. (DOCX 42 kb) [file 12917_2016_938_MOESM2_ESM.docx]

> Suid herpesvirus 1 isolate SuHV1/Lynx_pardinus/Badajoz/Spain/2015 glycoprotein B gene, partial

gtgctgatcgtctcgggcacctccaccatgcgcacgtagctgtagtcctcgtagtacacgtacccgctccccagcttaaagtagcgccggtggttgccggtgcagggctcgatgaggtcgcgcgagatgaggagctcgttgtcgtc
